# Migration and Fate Specification in the Ventral Striatum

**DOI:** 10.1371/journal.pbio.0030226

**Published:** 2005-05-17

Like other complex organs, the brain develops in stages, under tight control of multiple genetic signals. The key to understanding brain development, therefore, lies in understanding what these signals are, where they act, and what their effects are. The SOX family of transcription factors has emerged as a major group of developmental regulators in the brain, but their exact roles are not well known. In this issue, Vasso Episkopou and colleagues show that the mouse gene *Sox1* is required for the differentiation and migration of neurons within a portion of the developing brain.

A major event in brain development is the emergence of the lateral ganglionic eminence (LGE), a bulbous protrusion whose neurons go on to form several vital brain structures, including the ventral striatum (VS), which is ultimately responsible for control of various aspects of motor, cognitive, and emotional functions. Several transcription factors have been shown to operate within the LGE precursors of the VS, but these are not present after these neurons have stopped dividing. To determine whether the protein SOX1 might be playing a role in these cells, the authors examined mice missing the SOX1 protein only in these cells. They found that the absence of the protein from these post-mitotic cells (cells after cell division) prevented normal development of two VS structures, the striatal bridges and the olfactory tubercles.[Fig pbio-0030226-g001]

**Figure pbio-0030226-g001:**
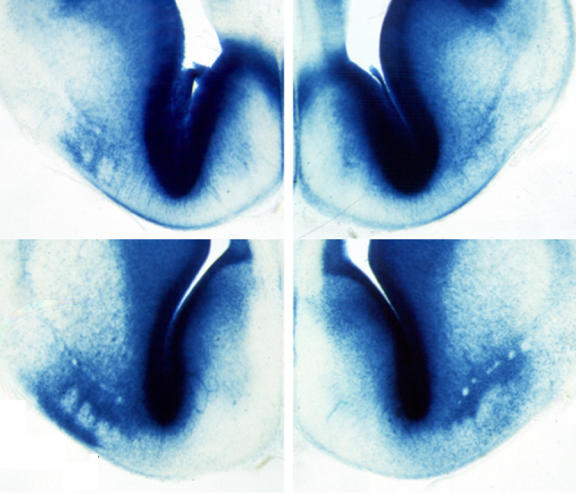
Sox1 gene expression in wild-type (left) and *Sox1* mutant (right) mouse forebrain

It appeared that the defect in mice missing SOX1 from all cells was not in the proliferation of neuronal precursors, which appeared normal, but instead in their differentiation and migration. And while the previously identified factors played no role after the neurons had stopped dividing, continued expression of *Sox1* in post-mitotic neurons was required for correct specification of cell identity. Overexpression of *Sox1*, on the other hand, did not increase the normal number of olfactory tubercle–specified neurons, or alter their migration, suggesting that regulation of these cells is under more complex layers of control.

These results, combined with previous work by the same authors showing a role for *Sox1* in terminal differentiation of the mouse lens, suggest that *Sox1* and other closely related family members may help specify and maintain final post-mitotic cell identity in a variety of tissue types.

